# Plant hormone induced enrichment of *Chlorella* sp. omega-3 fatty acids

**DOI:** 10.1186/s13068-019-1647-9

**Published:** 2020-01-17

**Authors:** Ramachandran Sivaramakrishnan, Aran Incharoensakdi

**Affiliations:** 1 Laboratory of Cyanobacterial Biotechnology, Department of Biochemistry, Faculty of Science, Chulalongkorn University, Bangkok, 10330 Thailand; 2 Academy of Science, Royal Society of Thailand, Bangkok, 10300 Thailand

**Keywords:** Docosahexaenoic acid, Eicosapentaenoic acid, Plant hormone, ROS, Antioxidant enzymes, Microalgae

## Abstract

**Background:**

Omega-3 fatty acids have various health benefits in combating against neurological problems, cancers, cardiac problems and hypertriglyceridemia. The main dietary omega-3 fatty acids are obtained from marine fish. Due to the pollution of marine environment, recently microalgae are considered as the promising source for the omega-3 fatty acid production. However, the demand and high production cost associated with microalgal biomass make it necessary to implement novel strategies in improving the biomass and omega-3 fatty acids from microalgae.

**Results:**

Four plant hormones zeatin, indole acetic acid (IAA), gibberellic acid (GBA) and abscisic acid (ABA) were investigated for their effect on the production of biomass and lipid in isolated *Chlorella* sp. The cells showed an increase of the biomass and lipid content after treatments with the plant hormones where the highest stimulatory effect was observed in ABA-treated cells. On the other hand, IAA showed the highest stimulatory effect on the omega-3 fatty acids content, eicosapentaenoic acid (EPA) (23.25%) and docosahexaenoic acid (DHA) (26.06%). On the other hand, cells treated with ABA had highest lipid content suitable for the biodiesel applications. The determination of ROS markers, antioxidant enzymes, and fatty acid biosynthesis genes after plant hormones treatment helped elucidate the mechanism underlying the improvement in biomass, lipid content and omega-3 fatty acids. All four plant hormones upregulated the fatty acid biosynthesis genes, whereas IAA particularly increased omega-3-fatty acids as a result of the upregulation of omega-3 fatty acid desaturase.

**Conclusions:**

The contents of omega-3 fatty acids, the clinically important compounds, were considerably improved in IAA-treated cells. The highest lipid content obtained from ABA-treated biomass can be used for biodiesel application according to its biodiesel properties. The EPA and DHA enriched ethyl esters are an approved form of omega-3 fatty acids by US Food and Drug Administration (FDA) which can be utilized as the therapeutic treatment for the severe hypertriglyceridemia.

## Background

The presence of double bond at the third carbon atom at methylene end in polyunsaturated fatty acids (PUFA) is a typical characteristic of omega-3 fatty acids. Human body cannot produce essential PUFA which can be obtained from various foods such as fish, plants and fish oils. General essential omega-3 fatty acids beneficial for humans are linolenic acid (C18:3), stearidonic acid (C18:4), eicosapentaenoic acid (C20:5), docosapentaenoic acid (C22:5) and docosahexaenoic acid (C22:6) [1]. Recent studies revealed that the omega-3-fatty acids have the beneficial effects on Alzheimer disease and dementia-like age-related diseases, cardiovascular complications and fetal development [2]. Low level of omega-3 fatty acids will cause mental problems such as depression, dyspraxia and dyslexia, neurodegenerative diseases and heart problems [2, 3]. The main limitation of the production of omega-3 fatty acids is raw materials. The current omega-3 fatty acid production is from fish which is being polluted with toxic heavy metals. An alternative production source relies on the use of microalgae which have a rapid growth under heterotrophic or stress conditions. *Chlorella* has been reported to be an important candidate for the commercial production of various pharmaceutical and nutraceutical products [4].

The overall production cost of omega-3 fatty acids is expensive and hence significant research on microalgae is required to cut the high production cost [5]. Plant hormones from the group of auxins play an important role in microalgal growth and metabolism [6]. For example, IAA plant hormone induces the cell division, biomass and pigment content in *Chlorella pyrenoidosa* and *Scenedesmus quadricauda* [7]. IAA also activates plasmalemmal ATPase which elongates the cells, increases cell plasticity, and alters the nucleic acid metabolism [8]. ABA is a plant hormone which can induce the production of biomass, carotenoids and lipids in *Euglena gracilis* [9]. Addition of exogenous cytokinins plant hormone induces the N signaling mechanism in microalgae and thereby enhances the biomass productivity [10]. Supplementation of another plant hormone gibberellic acid enhances the biomass growth and lipid content in the *C. sorokiniana* [11].

ROS is an important molecule which can regulate the development and growth of plants. The production of ROS in response to plant hormones treatment is one of plant hormones mechanisms to regulate the cellular growth and development of plants [12]. The block in ROS generation mechanism resulted in impaired root hair development and poor stress tolerance [13]. Auxin-induced plant growth regulation is associated with the ROS molecule, which is directly involved in cell wall loosening and stimulates antioxidant enzymes production [14]. Hence, it is necessary to analyze the cell’s ROS level, MDA (extent of damage caused by ROS) and antioxidant responses after the plant hormone treatment.

Most of the previous studies focused on improvement of lipid content in microalgae which can be utilized for the transesterification [15, 16]. However, there has been no information concerning the plant hormone-induced omega-3 fatty acids synthesis in microalgae. The aim of the present study is to enrich omega-3 fatty acids in *Chlorella* sp. treated with various plant hormones. The action of plant hormones in the cells was studied with various analyses including, biomass and lipid content, antioxidant markers and antioxidant enzymes, and fatty acid composition determination.

## Results and discussion

### Effect of plant hormones on *Chlorella* sp. biomass production

**Fig. 1:**
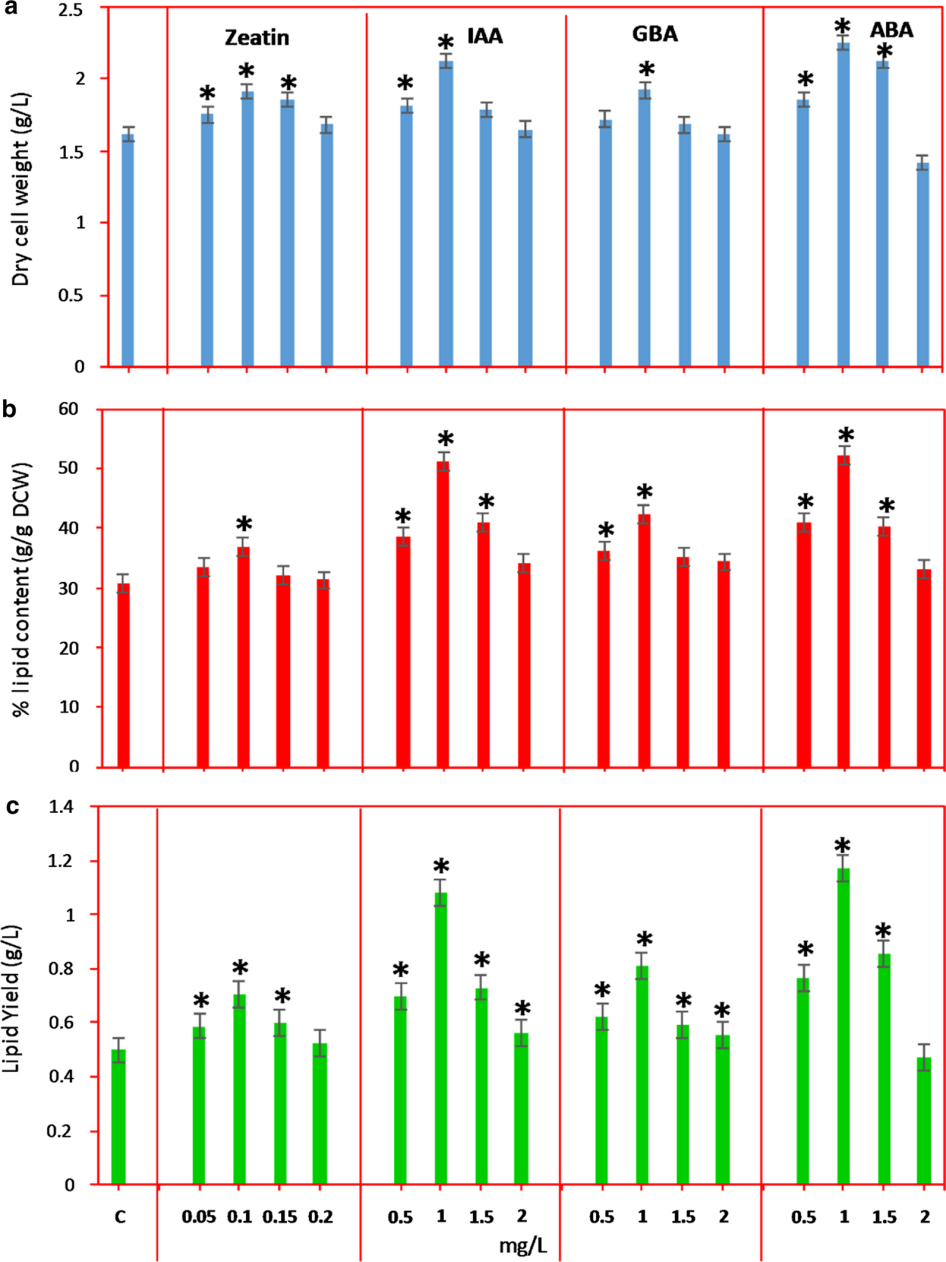
Effect of different concentrations of phytohormones zeatin, IAA, GBA and ABA on **a** biomass **b** lipid content and **c** lipid yield in 12 days grown cells (conditions: modified BG-11 medium at 27 °C ± 1). Data points were from three independent experiments with error bars showing standard deviation, (*) represents the statistical significance with respect to control values at *p* < 0.05. The label c on the X-axis represents the control

Different plant hormones such as zeatin, IAA, gibberellic acid and abscisic acid were used to examine their effects on the biomass content of *Chlorella* sp. (Fig. 1a). All the hormones concentrations tested, except at 2 mg/L, increased the biomass content. Among the four hormones, treatment with ABA showed highest biomass content. Zeatin, IAA and gibberellic acid treated cells showed similarly increased biomass content. In all the cases with different hormones treatment, except zeatin, maximum biomass was obtained with the addition of 1 mg/L of hormone to the growth medium. In case of zeatin, the maximum biomass was obtained with 0.1 mg/L of hormone supplementation.

Zeatin plays an important role in cell growth response and embedding of the lipids. Supplementation of zeatin stimulates the key factors and enzymes involved in nitrogen metabolism which improves the growth and cell division [17]. Moreover, supplementation of zeatin stimulates the activity of ribulose 1,5-bisphosphate carboxylase/oxygenase and protein synthesis as evident by the increase in chlorophyll, growth and carbohydrate content in plant leaves [18].

Another important phytohormone is gibberellic acid which improves the growth, metabolism and development of plants and algae. A study by Aftab et al. [19] reported that gibberellic acid is the promising phytohormone which can stimulate the growth, stomatal conductance and photosynthesis in sweet wormwood. Under nitrogen limitation, gibberellic acid also stimulates the activity of the carbonic anhydrase, nitrate reductase and key enzymes involved in the carbon and nitrogen metabolism.

Auxin (IAA) plays an important role in cell growth and metabolism. A very low concentration of auxins induces the biomass production and improves the biosynthesis of valuable biological molecules [20]. A study in *Scenedesmus* sp. showed that the supplementation of IAA increased the biomass by 1.9-fold [21]. In another study, auxin addition resulted in a significant increase of the biomass content by 2.2-fold in *C. pyrenoidosa* [22]. In addition, supplementation of auxin stimulates the photosynthesis activity by enhancing the chlorophyll content and activates the cellular redox systems [23]. Udayan et al. [24] reported a 2.5-fold increase of the cell number in *Nannochloropsis oceanica* CASA CC201 after the supplementation of 10 ppm IAA.

ABA also plays an important role in triggering cell growth and metabolic activities. However, the addition of ABA was shown to decrease the growth of marine diatoms like *Coscinodiscus granii* [25] and *Nannochloropsis oceanica* [26]. In the present study, ABA highly stimulated the growth of *Chlorella* sp. as compared to other plant hormones (Fig. 1a). In *S. quadricauda*, treatment with ABA improved the biomass yield by 1.4-fold when compared with that under nitrogen starvation [27]. ABA also acted as an effective regulator and influenced the cell growth and developmental processes [27].

### Growth kinetics of *Chlorella* sp. after hormone treatment

**Fig. 2:**
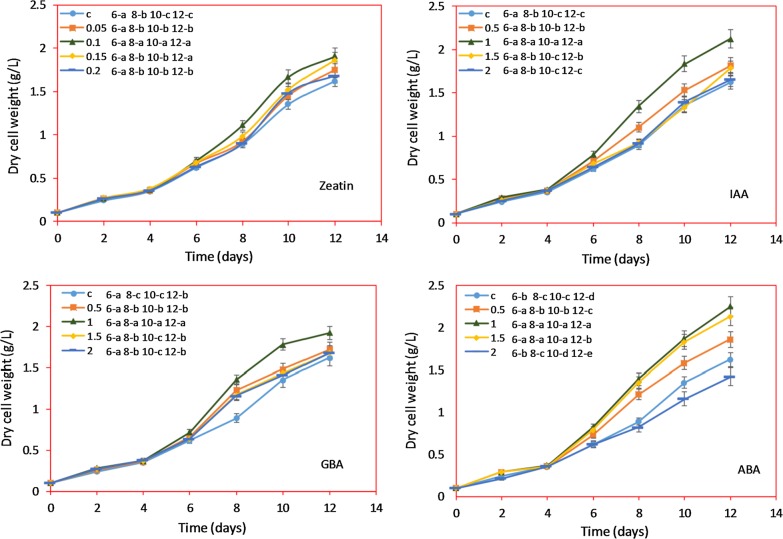
Growth kinetics of *Chlorella* sp. on different plant hormone supplementations (conditions: modified BG-11 medium at 27 °C ± 1). Data points were from three independent experiments with error bars showing standard deviation. The different letters (maximum value is ‘a’) indicate significant differences between different plant hormone concentrations at day 6, 8, 10 and 12 (*p* < 0.05)

After the addition of hormones, biomass concentration and growth kinetics were determined. The data presented in Fig. 2a–d showed that the growth of *Chlorella* sp. started to spike at 4th day. The significant increase in biomass was found until 12th day, beyond which the biomass was not significantly increased and reached the stationary phase (data not shown). In all cases (hormone treatment conditions and control) the trend of algal growth was similar. However, the biomass was varied depending on the hormone type used. The maximum biomass concentration was achieved with ABA and IAA-treated cells. The growth rate and speed of hormones action (doubling time) was determined according to the growth kinetics data (Table 1). In all the four hormone (zeatin, IAA, GBA and ABA) treated samples, the maximum growth rate was achieved at 0.1, 1, 1, and 1 mM concentration, respectively. The zeatin-treated *Chlorella* sp. had maximum growth rate of 0.273 ± 0.17/day and satisfactory doubling time of 1.989 ± 0.14 day at 0.1 mM concentration. In case of IAA, the maximum cell growth rate of 0.286 ± 0.23/day was achieved at 1 mM concentration with 1.94 ± 0.11 day doubling time. For GBA, the maximum growth rate of 0.274/day and 1.986 ± 0.15 day doubling time at 1 mM concentration was observed. The highest growth rate of 0.300 ± 0.23/day and satisfactory doubling time of 1.894 ± 0.14 day were obtained with 1 mM ABA treatment. Chia et al. studied the *C. vulgaris* growth in different culture media and the maximum growth rate was found to be 0.14/day [28]. In the present study, the growth rates of IAA and ABA-treated cells were highest which indicate an obvious effectiveness of these two plant hormones for improving the biomass of *Chlorella* sp.

**Table 1:** Maximum growth rate (µ_max_) of *Chlorella* sp. after the plant hormone supplementation and its doubling time

Hormone concentration (mg/L)	µ_max_/day	Doubling time (in days)
Control	0.255375 ± 0.16	2.058171 ± 0.11
Zeatin		
0.05	0.263545 ± 0.15	2.02668 ± 0.13
0.1	0.273559 ± 0.17	1.989384 ± 0.14
0.15	0.264694 ± 0.18	2.02233 ± 0.12
0.2	0.256741 ± 0.21	2.052835 ± 0.13
IAA		
0.5	0.264597 ± 0.18	2.022696 ± 0.12
1	0.2865 ± 0.23	1.943164 ± 0.11
1.5	0.261811 ± 0.21	2.03328 ± 0.14
2	0.249171 ± 0.19	2.082762 ± 0.11
GBA		
0.5	0.260663 ± 0.21	2.037675 ± 0.14
1	0.27443 ± 0.22	1.986208 ± 0.15
1.5	0.252174 ± 0.19	2.070782 ± 0.13
2	0.24773 ± 0.2	2.088564 ± 0.13
ABA		
0.5	0.273705 ± 0.15	1.988853 ± 0.13
1	0.300864 ± 0.23	1.894245 ± 0.14
1.5	0.296296 ± 0.22	1.909545 ± 0.13
2	0.228718 ± 0.21	2.168413 ± 0.13

### Effect of plant hormones on lipid content and yield

Some recent studies revealed that the supplementation of phytohormones causes embedding of high lipid content and other high-value products [20, 29]. Enhancing lipid and biomass contents was the ultimate aim of this study. Addition of plant hormones to the medium elevated the lipid content in all cases (Fig. 1b). The treatment with zeatin showed the lowest lipid content among the four hormones tested. Zeatin at 0.1 mg/L embedded the maximum lipid content in the cells when compared to other concentrations of the zeatin. A further increase in the zeatin concentration decreased the lipid content gradually, but the content was still higher than the control. IAA and ABA treatment produced the highest lipid content at 1 mg/L concentration which is the highest among all the tested plant hormones concentrations. The 60.9% of lipid content was observed in the *Nannochloropsis oceanica* CASA CC201 after the addition of 40 ppm IAA which was 2-fold higher than the control (31.05%) [24]. In the present study, IAA and ABA-treated cells showed 51 and 52% lipid content, a 20% and 21% increase, respectively. Gibberellic acid treatment showed stimulatory effect on lipid content when compared to zeatin; however, the lipid content in gibberellic treated *Chlorella* was lower than those treated with ABA and IAA. Addition of phytohormones like gibberellic acid resulted in a 2-fold increase in biomass and a 3-fold increase in neutral lipid of a freshwater diatom [30]. In the present study, gibberellic acid treatment resulted in 12% increase in the lipid content of *Chlorella*. This suggested that the response of cells to gibberellic acid is species dependent.

The addition of plant hormones increases the glucose utilization rate in *Aurantiochytrium* sp. YLH70 and their metabolites were increased in mevalonate pathway and fatty acid biosynthesis pathway [31]. On the other hand, glycolysis and TCA cycle metabolites were decreased. Addition of plant hormone increased the protein and lipid content rapidly from 31.4 to 39.7% and from 44.6 to 54.3%, respectively, in *Monoraphidium* sp. FXY-10 [29]. In addition, plant hormone upregulated the malic enzyme (ME), acyl CoA carboxylase D and glycerol-3-phosphate acyltransferase expressions in *Monoraphidium* sp. FXY-10, whereas it downregulated the phosphoenol pyruvate carboxylase (PEPC). Li et al. [32] reported that the combination of plant growth regulator (melatonin) and high light intensity elevated the 1.32-fold lipid accumulation in *Monoraphidium* sp. QLY with no change in biomass content, whereas plant growth regulator addition alone increased both the acyl CoA carboxylase and ME levels and decreased the PEPC level. The addition of plant growth regulator (melatonin) increased the lipid biosynthetic genes expression levels leading to the increase in lipid content [33]. It is thus likely that the improved lipid content in *Chlorella* sp. after the addition of plant hormones is a result of the upregulated lipid biosynthetic genes expression.

The overall lipid production with respect to biomass content was determined and the results showed that the maximum lipid yields of 1.08 and 1.17 g/L were achieved upon treatment with the 1 mg/L IAA and ABA, respectively (Fig. 1c) The overall lipid yield was increased due to the combined increase of biomass (Fig. 1a) and lipid content (Fig. 1b) after the addition of 1 mg/L IAA and ABA.

Phytohormones protect the enzymes involved in the biosynthesis of pigments and lipids which can also be affected by other factors. Plant hormones are found to improve the photosynthetic efficiency and CO_2_ fixation in microalgae [16]. Plant hormones, especially auxins nitrogen group, highly stimulate the Calvin cycle with the increase in chlorophyll content. Nitrogen compounds are important components for cellular growth and metabolism in prokaryotic and eukaryotic cells [34].

The report by Piotrowska et al. [35] showed that the cellular DNA level was increased up to 48% and it reflects in the protein synthesis where up to 20–40% of soluble protein content was improved. The proteins may be pigment protein complexes and other enzymes which are stimulated by the plant hormones. The present study agreed with that of Piotrowska et al. [35] since the chlorophyll and carotenoids were elevated after the hormone treatment.

### Effect of different plant hormones on fatty acid profiles

All the plant hormones used in this study elevated the lipid contents in the *Chlorella* sp. (Fig. 1b). Different plant hormones affected the alga differently in terms of fatty acid composition, i.e., fatty acid compositions were altered when treated with different plant hormones (Table 2). The ABA treatment showed highest biomass and total lipid content (Fig. 2a, b). However, in case of fatty acid profiles IAA and ABA treatment caused higher variation of fatty acid compositions than did zeatin and GBA treatment (Table 2).

**Table 2:** Fatty acid composition of plant hormone-treated *Chlorella* sp.

Hormone concentration mg/L	Fatty acids (%)									
	Myristic acid	Palmitic acid	Palmitoleic acid	Stearic acid	Oleic acid	Linoleic acid	Linolenic acid	Arachidic acid	Eicosapentaenoic acid	Docosahexaenoic acid
Control	0.24 ± 0.01	29.37 ± 1.03	12.46 ± 0.3	2.02 ± 0.12	0.22 ± 0.01	24.17 ± 1.21	31.52 ± 1.42	0 ± 0.0	0 ± 0.0	0 ± 0.0
Zeatin										
0.05	0.25 ± 0.02	28.33 ± 1.3	12.77 ± 0.42	1.51 ± 0.09	0.12 ± 0.01	21.1 ± 1.32	31.61 ± 1.33	2.26 ± 0.13	2.05 ± 0.13	0 ± 0.0
0.1	0.24 ± 0.01	27.13 ± 1.2	12.16 ± 0.51	1.22 ± 0.11	0 ± 0.0	20.11 ± 1.16	33.12 ± 1.32	3.21 ± 0.14	2.81 ± 0.09	0 ± 0.0
0.15	0.22 ± 0.03	28.11 ± 1.01	12.78 ± 0.66	1.43 ± 0.13	0.11 ± 0.01	20.11 ± 0.94	31.12 ± 1.37	3.24 ± 0.21	2.88 ± 0.14	0 ± 0.0
0.2	0.21 ± 0.01	28.12 ± 1.4	12.71 ± 0.52	1.21 ± 0.11	0.11 ± 0.01	20.12 ± 1.03	31.54 ± 1.28	3.21 ± 0.13	2.77 ± 0.1	0 ± 0.0
IAA										
0.5	0.25 ± 0.02	19.33 ± 1.02	11.65 ± 0.41	1.43 ± 0.08	0.21 ± 0.02	20.1 ± 0.94	24.61 ± 1.09	2.25 ± 0.14	9.05 ± 0.23	11.12 ± 0.56
1	0.24 ± 0.01	16.73 ± 1.03	8.76 ± 0.53	1.02 ± 0.09	0 ± 0.0	10.11 ± 0.46	12.67 ± 0.65	1.11 ± 0.09	23.25 ± 0.43	26.11 ± 0.54
1.5	0.23 ± 0.01	16.71 ± 1.3	8.68 ± 0.32	1.01 ± 0.99	0 ± 0.0	10.11 ± 0.51	12.71 ± 0.78	1.11 ± 0.08	23.31 ± 1.71	26.13 ± 0.47
2	0.23 ± 0.01	16.92 ± 1.24	9.29 ± 0.65	1.02 ± 0.10	0 ± 0.0	10.02 ± 0.63	12.12 ± 0.53	1.03 ± 0.1	23.31 ± 1.83	26.06 ± 0.48
GBA										
0.5	0.25 ± 0.01	28.33 ± 1.23	12.77 ± 0.91	1.51 ± 0.14	0.12 ± 0.01	28.71 ± 1.12	28.31 ± 1.42	0 ± 0.0	0 ± 0.0	0 ± 0.0
1	0.24 ± 0.03	28.11 ± 1.08	11.23 ± 0.82	1.14 ± 0.91	0.13 ± 0.02	32.07 ± 1.27	27.08 ± 1.23	0 ± 0.0	0 ± 0.0	0 ± 0.0
1.5	0.24 ± 0.05	28.12 ± 1.32	11.18 ± 0.83	1.17 ± 0.82	0.16 ± 0.01	33.11 ± 1.21	26.02 ± 1.52	0 ± 0.0	0 ± 0.0	0 ± 0.0
2	0.24 ± 0.04	28.13 ± 1.35	11.18 ± 0.62	1.16 ± 0.9	0.16 ± 0.02	33.12 ± 1.33	26.01 ± 1.07	0 ± 0.0	0 ± 0.0	0 ± 0.0
ABA										
0.5	0.24 ± 0.02	26.72 ± 1.24	24.12 ± 1.02	1.04 ± 0.08	0.31 ± 0.03	22.38 ± 1.03	21.91 ± 1.04	0.13 ± 0.02	2.04 ± 0.11	1.11 ± 0.11
1	0.24 ± 0.02	24.42 ± 1.32	24.63 ± 0.98	1.03 ± 0.07	0.31 ± 0.02	23.07 ± 1.04	22.03 ± 0.94	0.13 ± 0.01	3.02 ± 0.13	1.12 ± 0.12
1.5	0.24 ± 0.04	24.42 ± 1.24	24.71 ± 0.93	1.01 ± 0.06	0.31 ± 0.02	22.99 ± 1.05	22.02 ± 1.03	0.12 ± 0.01	3.01 ± 0.15	1.17 ± 0.13
2	0.24 ± 0.03	24.41 ± 1.04	24.7 ± 1.04	1.01 ± 0.05	0.32 ± 0.02	23.02 ± 1.06	22.01 ± 1.02	0.12 ± 0.01	3.01 ± 0.13	1.16 ± 0.12

Supplementation of zeatin increased the long chain fatty acids in the cells which are arachidic acid 2.26% and eicosapentaenoic (EPA) acid 2.05% (Table 2). The untreated cells already contained linolenic acid and the addition of zeatin induced the occurrence of arachidic acid and EPA. Both linolenic and EPA are omega-3 fatty acids. It is worth noting that the docosahexaenoic acid (DHA), in addition to arachidic acid and EPA, was improved after treatment with IAA. DHA is an important component of the omega-3 fatty acids. The addition of IAA improved the growth considerably and enhanced the 56% of polyunsaturated fatty acid content in *S. obliquus* GU732418 [21]. The linolenic acid (omega-3-fatty acid) content was increased from 7.62 to 17.49% [21]. Jusoh et al. [36] reported that the addition of IAA to the growth media decreased the palmitic, stearic acids and linoleic acids content and simultaneously increased the other fatty acids contents in *C. vulgaris.* The 40 ppm supplementation of IAA elevated EPA content from 1.87 to 10.76% in *Nannochloropsis oceanica* CASA CC201 [24]. In the present study, IAA at 1 mg/L produced 12.67% of linolenic acid, 23.25% of EPA and 26.11% of DHA, and overall PUFA production after IAA supplementation was 63.14%, a 2-fold increase compared to the control (31.52%).

The PUFA concentration *in C. vulgaris* was significantly increased about 146.81% after 10 days of IAA treatment [36]. The expression of ω-6 fatty acid desaturase (FAD) gene in *C. vulgaris* was also shown to increase after 6 days of IAA treatment [36]. This was reflected in fatty acid composition which is rich in PUFA content. Stearns and Morton [37] reported that the auxin treatment increased the saturated fatty acid content and decreased the PUFA content in soybean. On the other hand, Liu et al. [38] reported that the C18:2 content was decreased and C18:3 content was increased in soybean zygotic embryo cotyledons after the auxin treatment. In the present study, the PUFA content was high at 12th day after IAA treatment. Previously, PUFA was suggested to be the main fatty acid in triglycerides of *C. vulgaris* after the IAA treatment [36]. Moreover, PUFA content in algal cells was elevated during late log phase in conjunction with an alteration in fatty acid composition upon plant hormone treatment [36]. In the present study, IAA treatment altered the PUFA content in fatty acid composition with the occurrence of arachidic acid, EPA and DHA content which were absent in the untreated control. However, zeatin and ABA treatment also showed small amount of omega-3 fatty acid content which was much lower than that found with IAA treatment. In addition, an enhanced photosynthetic rate by plant hormone treatment (Additional file 1) could induce oxidative stress due to H_2_O_2_ production, which led to the increase in lipid and PUFA content.

Hence it is clear from the previous studies, that the IAA treatment enhances the PUFA content by enhancing the expression of genes responsible for PUFA synthesis [36–38]. The cells could produce new fatty acids which were not found in the control during stress conditions. Mohan and Devi [39] studied the lipid content of microalgae after salt stress and observed the variations in fatty acid compositions. The saturated fatty acid was increased and arachidic acid, which was absent in the control, was found in the fatty acid compositions after salt stress [39].

On the other hand, DHA and EPA production was less stimulated by ABA treatment when compared to IAA treatment (Table 2). Du et al. [40] studied the fatty acid desaturation and lipid content after ABA treatment on *C. pyrenoidosa*, which showed the absence of linoleic acid and arachidonic acid and a decreased PUFA content, as well as the reduced fatty acid desaturase expression. ABA treatment in the present study also resulted in very low PUFA content in *Chlorella* sp. (Table 2). Similarly, other plant hormones showed higher fatty acid desaturase expression than did the ABA treatment [40]. In addition, plant hormones could stimulate fatty acid elongation process. The highest carbon chain length fatty acid appearing in *C. pyrenoidosa* was C20:4, whereas plant hormone treatment increased the carbon chain length with the occurrence of new fatty acid C22:4 in the fatty acid profile. The expression of several genes related to fatty acid metabolism was differentially affected by different plant growth regulators in microalgae including fatty acid elongation [40]. Azachi et al. [41] studied the induction of fatty acid elongase in the microalgae *Dunaliella salina* and found that the salt stress induced the elongation and desaturase which increased the carbon chain length and desaturation of fatty acids. Similar to salt stress, oxidative stress created by plant hormone in the present study also increased the carbon chain length and desaturation. The results in the present study were in agreement with those of Du et al. [40] which showed that the plant hormone treatment and stress created by plant hormone especially IAA increased the carbon chain length and desaturation.

In case of gibberellic acid treatment which did not improve the omega-3 fatty acid content, it showed variations in the contents of linoleic and linolenic acid. Slight variations were observed with palmitic, palmitoleic, stearic and oleic acid contents. Hence from the present study, it is clear that the different concentration of gibberellic acid did not show any significant variations in the fatty acid compositions.

Different ABA concentrations caused high variations in the contents of palmitoleic, oleic, linoleic and linolenic acid and considerable variations were observed with palmitic acid. ABA also induced arachidic acid, EPA and DHA production, but in very small amount with mild variations in other fatty acids.

The fuel properties obtained from ABA-treated *Chlorella* sp. were within acceptable range values mentioned in American Society for Testing and Materials and European standards. Hence ABA supplementation can be a promising strategy to achieve the high lipid content and biomass for the transesterification which is adopted for the biodiesel production.

The overall lipid content is high with ABA treatment, but the instance of high omega-3 fatty acid content can be achieved with IAA treatment which is very valuable when compared to the other plant hormones.

Hypertriglyceridemia is a common threat to the world, which can cause atherosclerosis (cardiac disease) and acute pancreatitis. Lowering the triglyceride is the primary treatment to reduce the hypertriglyceridemia. It can be achieved by therapeutic intake of fibrates, omega-3 fatty acids and nicotinic acid. The ingestion of omega-3 fatty acids including EPA, DHA which are the important components was found to markedly reduce the triglyceride concentration [42]. The US Food and Drug Administration has approved various omega-3 fatty acids for treatment of hypertriglyceridemia.

#### Mechanism involved in the action of plant hormones

**Fig. 3:**
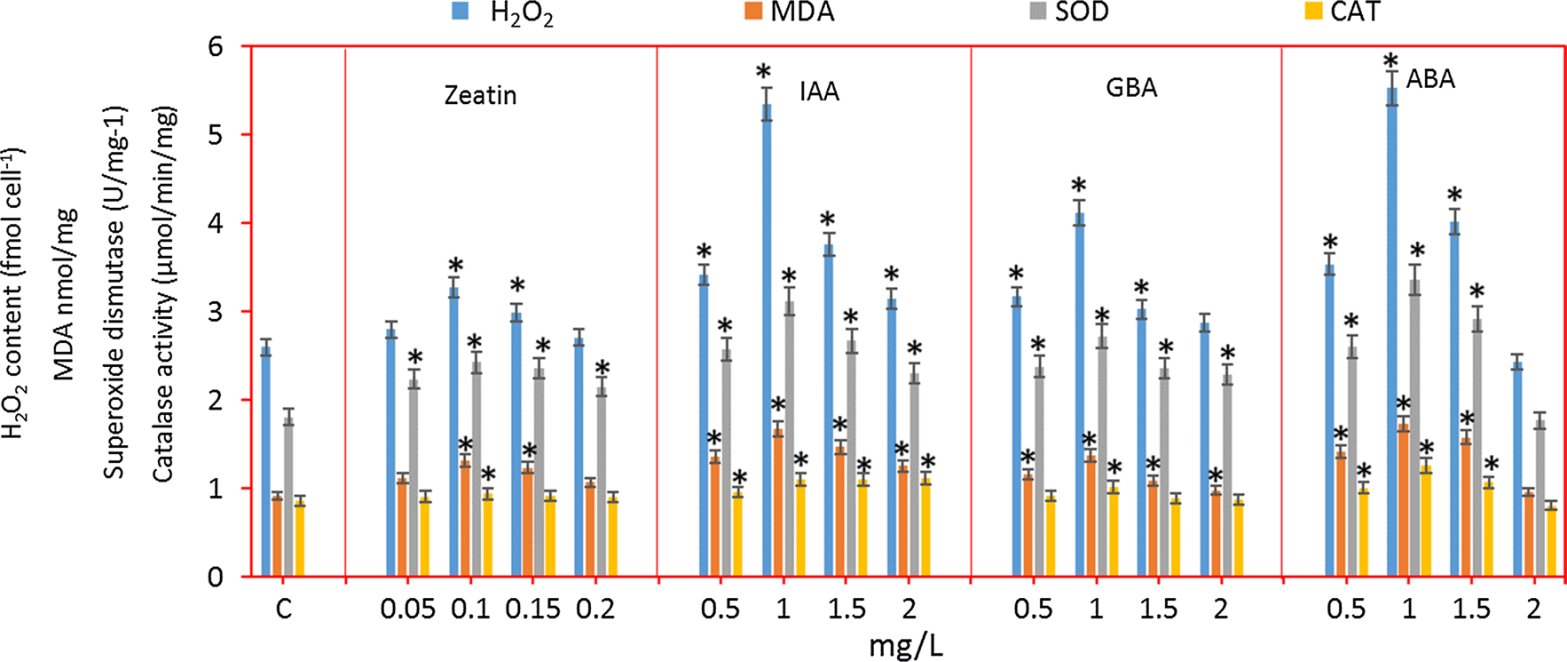
H_2_O_2_ and MDA levels, SOD and CAT activities on different plant hormone supplementations. Data points were from three independent experiments with error bars showing standard deviation, (*) represents the statistical significance with respect to control values at *p* < 0.05. The label c on the X-axis represents the control

After the addition of plant hormones, significant improvement in growth and lipid content was observed. The analysis of ROS compounds and enzymes helps to reveal the mechanism underlying the improvement of cell growth and lipid content. H_2_O_2_ content was determined after the hormone treatment and shown in Fig. 3. H_2_O_2_ is an important marker to assess the level of ROS generation. The results showed that the addition of plant hormones increased the H_2_O_2_ content in all cases. In case of zeatin, H_2_O_2_ generation is high at 0.1 mM zeatin. The hormones IAA and ABA at 1 mM concentration exhibited high H_2_O_2_ levels. The addition of exogenous hormones increased the H_2_O_2_ content, whereas addition of cytokines (zeatin) showed low H_2_O_2_ production and addition of auxins showed high H_2_O_2_ levels. The ROS promotes cell differentiation and proliferation by acting as a signaling molecule which improves the cell growth and physiological responses [43].

The H_2_O_2_ production in the present study is possibly occurring in chloroplast as reported by Mehler [44]. In the present study, plant hormone induces the high photosynthetic activity which is evident by the increased oxygen evolution rate. The increase in oxygen evolution results in excess O_2_ availability which is later reduced leading to the generation of H_2_O_2_ [44]. On the other hand, high photosynthetic efficiency increases the O_2_^−^ which is further reduced by Fe–S centers in chloroplast electron transport system and generates H_2_O_2_ [45].

Many plant hormones play a vital role in the regulation of plant cells. Plant hormones protect the photosynthetic apparatus and chloroplast structure [46]. Auxin increased the photosynthesis rate and induced maximal electron transport rate [47]. The *Chlorella* sp. in the present study showed increased Chl-*a* content (Additional file 1: Fig. S2a) which is the evidence that H_2_O_2_ does not affect the chloroplast.

The MDA is another important marker to determine the damage caused by ROS. The MDA level was elevated after the addition of hormones in all cases (Fig. 3). The maximum MDA value was observed with the IAA and ABA at 1 mM concentration. It is clear, that the addition of IAA and ABA led to more ROS generation which slightly damaged the cell and improved the MDA content. The levels of SOD and CAT, which are important antioxidant enzymes, were elevated after the addition of hormones. This suggested that the plant hormone-treated *Chlorella* sp. can fight against the ROS molecules (Fig. 3). The maximum SOD and CAT activity was found with the 1 mM IAA and ABA. The ROS generation creates oxidative stress to the cells. The oxidative stress in turn improves the antioxidant enzymes levels. The SOD is categorized into the metalloenzyme which acts as a superoxide scavenger [48]. Plant hormones act as a growth hormone which stimulates the development of plant growth [49]. Plant hormones improve the cell viability and play an important role in the regulation of plant cells differentiation. The action of plant hormones on microalgae is similar to that of higher plants [50].

During the treatment of cells with plant hormones, soluble proteins levels were increased, whereas monosaccharide levels were decreased. In the present study, the total protein content was improved after the addition of plant hormones (data not shown). The increased soluble proteins indicated the improved metabolic and mitotic activity leading to the promotion of the cell growth [43]. When algal cells were treated with the plant hormone, carotenoids levels were increased which play a protective role against ROS molecules by absorbing more visible light and protect the chloroplasts against photoinhibition [43]. The increased carotenoids from the present study (Additional file 1: Fig. S2b) indicates that the *Chlorella* sp. can cope with the ROS molecules formed during plant hormone treatment. The overproduction of plant hormones by transgenic tobacco increased the activities of SOD and CAT which contribute to the defense mechanism against ROS by protecting the photosynthetic apparatus from degradation [51]. The addition of exogenous IAA induces the SOD, CAT and peroxidases activities which protect the *Triticum aestivum* L. cells [52]. The exogenous addition of plant hormones stimulates the biomass and lipid content in *C. protothecoides* [53].

Previous studies reported the improvement of cell growth and lipid content after the addition of the plant hormones. However, in the present study, it is worth noting that the PUFA content in the lipid was markedly enriched upon the addition of IAA plant hormone (Table 2). Exogenous addition of IAA improves the expression of desaturase in *C. vulgaris*, particularly the genes responsible for the synthesis of omega-3 fatty acids [36]. The synthesis of lipid requires more energy when compared with carbohydrate and protein synthesis. During oxidative stress the ROS-derived electrons are utilized for lipid synthesis and thus reducing the damages caused by ROS [54]. Moreover, stress-mediated redox-sensitive enzymes can regulate the key metabolic pathways like lipid synthesis [55].

In the present study, addition of plant hormones induces the ROS which was confirmed by the elevation of oxidative stress markers (H_2_O_2_ and MDA). The algal cell itself produces antioxidant enzymes to protect the cell from oxidative stress. The oxidative stress created by ROS improves the cell proliferation and differentiation [54, 56].

H_2_O_2_ is an oxidant molecule which causes stress to the cells. However, hormone treatment protects the cells from severe damage. It is clear from the previous report that plant hormone treatment protects the chloroplasts and its reaction center [45]. During stress conditions the photosynthetic organism embeds more unsaturated fatty acids [57]. In the present study, the plant hormone treatment increased the oxygen evolution rate (Additional file 1: Fig. S2c) indicating increased photosynthesis, which in turn increased H_2_O_2_ production. On the other hand, plant hormone treatment could protect the chloroplasts as evident by the increased chl-*a* content (Additional file 1: Fig. S2a). In addition, the cells also responded against the ROS molecule by increasing the activity of the antioxidant enzymes (Fig. 3). It is clear from the previous studies that the oxidative stress increased the growth rate and lipid production [58, 59]. During ROS generation, cells could counteract ROS by accepting ROS-mediated electron in the lipid synthesis pathway to nullify the ROS effects [54]. The enzymes in lipid synthesis pathway are highly redox sensitive, hence during ROS generation lipid biosynthesis pathway enzymes and carbon assimilation pathway enzymes are highly regulated to embed more lipids [55].

### ACC activity and transcriptional expression analysis of fatty acid synthesis-related genes

**Fig. 4:**
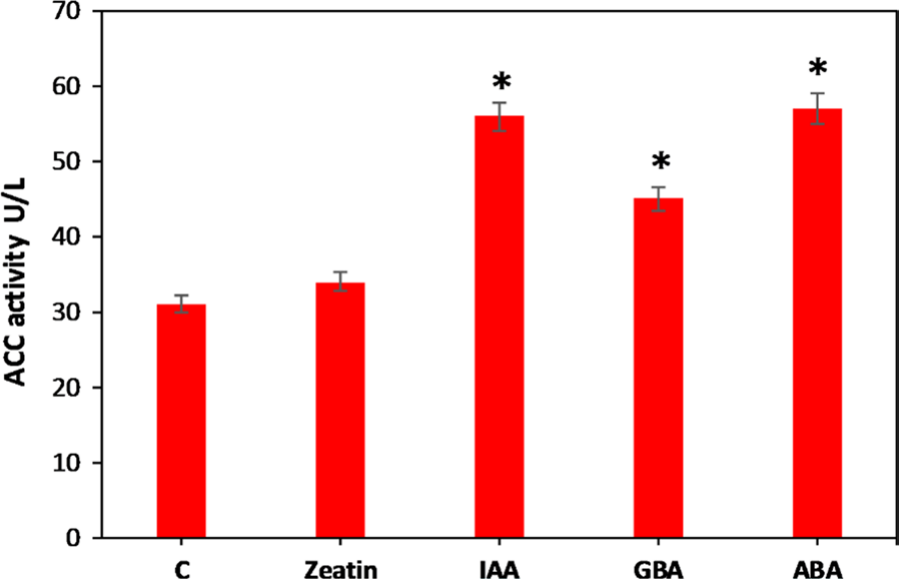
Effect of plant hormone treatment on ACC activity. Data points were from three independent experiments with error bars showing standard deviation, (*) represents the statistical significance with respect to control values at *p* < 0.05. The label c on the X-axis represents the control

**Fig. 5:**
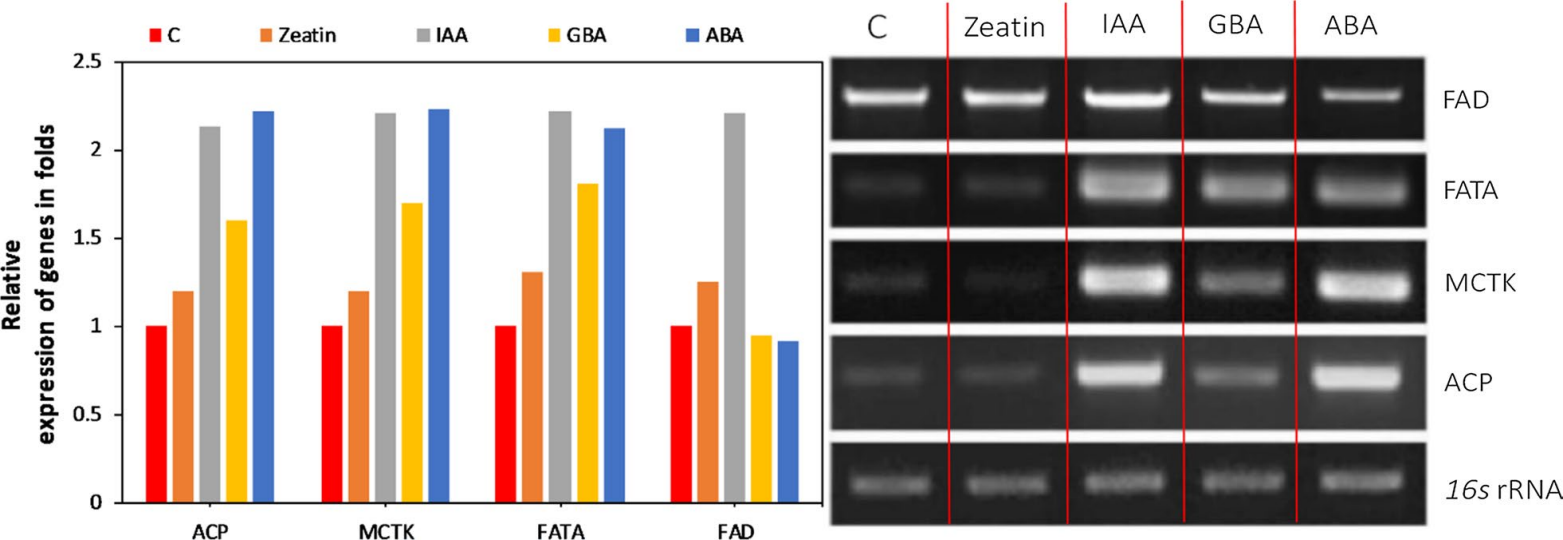
Relative transcript levels of *acp*, *mctk*, *fata* and *fad* of plant hormone-treated *Chlorella* sp. The label c represents the control

**Fig. 6:**
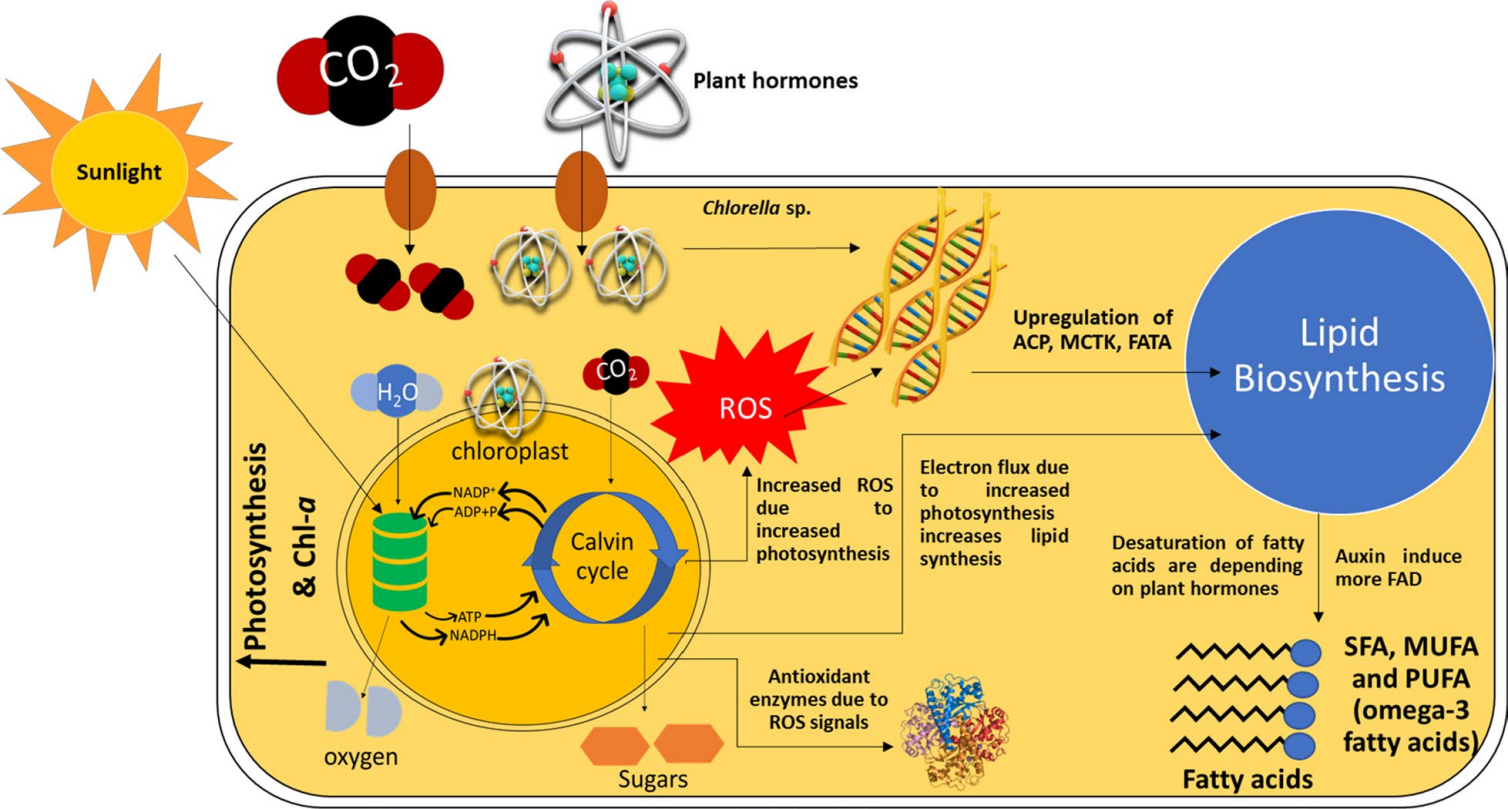
Mechanistic model of the omega-3 fatty acid production induced by plant hormones

The total fatty acid content of *Chlorella* sp. was improved after treatment with all four plant hormones. In addition, omega-3 fatty acids were highly increased with the IAA addition. It is clear that the plant hormone-induced oxidative stress increased the fatty acid content. However, it is important to understand the mechanism underlying the regulation of fatty acid biosynthesis in plant hormone-treated *Chlorella* sp. Hence, the acetyl-CoA carboxylase (ACC) activity, which is involved in the first committed step for the fatty acid biosynthesis, of each plant hormone-treated samples was determined. Moreover, the expression levels of other important genes which are involved in the fatty acid biosynthesis pathway, i.e., acyl–acyl carrier protein (*acp*), malonyl-CoA:ACP transacylase (*mctk*), acyl carrier protein thioesterase (*fata*) and omega-3 fatty acid desaturase (*fad*) were quantified using RT-PCR. The ACC activity after different plant hormone treatment is shown in Fig. 4. Zeatin showed lowest ACC activity when compared to other plant hormones. There was no significant difference between control and zeatin treated. However, IAA and ABA showed similarly high ACC activity, indicating the significant improvement of fatty acid synthesis in *Chlorella* sp. after the IAA and ABA treatment. GBA treatment showed a slight increase of ACC activity compared to the control and zeatin treatment. The plant hormone treatment increased the ACC activity in all cases with particularly high activity under IAA and ABA treatment. Figueroa-Balderas et al. [60] reported that the ACC activity was increased by plant hormone mediated stress. Similarly, in the present study, the two plant hormones IAA and ABA showed highest ROS levels in conjunction with the highest ACC activity (Figs. 3 and 4). The expression of important genes involved in fatty acid synthesis pathway upon plant hormone treatment was determined and shown in Fig. 5. Among the different plant hormones, fatty acid biosynthesis genes were highly activated by IAA and ABA. *acp*, *mctk* and *fata* were highly upregulated by IAA and ABA, whereas zeatin and GBA showed little or no upregulation. On the other hand, *fad* was highly upregulated by IAA, whereas other hormones did not show significant difference when compared with the control. The genes involved in the fatty acid synthesis were upregulated under plant hormone treatment and in the present study, *acp*, *mctk*, *fata* and *fad* showed upregulation after plant hormone treatment. In addition, IAA treatment showed high *fad* expression level corresponding to the increase of omega-3 fatty acids production (Table 2). Lei et al. [61] reported that the various fatty genes were upregulated under stress condition and showed high fatty acid content in *Haematococcus pluvialis*. In another study, mRNA levels of fatty acid biosynthesis genes in *H. pluvialis* were upregulated by plant hormones [62]. Lin et al. [63] studied the effect of plant growth regulators on fatty acid synthesis genes transcription level in *C. vulgaris* and found that the fatty acid synthesis genes were upregulated after ABA treatment and showed high omega-3 fatty acid content. However, in the present study, isolated *Chlorella* sp. showed high omega-3 fatty acid content after the IAA treatment. The mechanism of the plant hormone action according to the obtained results is shown in Fig. 6.

### Economical assessment of plant hormones treatment

It is necessary to justify the cost of plant hormones treatment used to improve the biomass and lipid content on the economical aspect. Plant hormones are generally required in a very small amount to improve the biomass and valuable materials in microalgae. Salaama et al. [21] summarized the economic estimation of the use of phytohormones for microalgal biomass production. The biomass production with IAA supplementation was more economical than that without the phytohormone.

Singh et al. [64] reported that the very small amounts of plant hormones such as diethyl amino ethyl hexanoate (DAH) and IAA (1.75–2.15 mg/L) were required to increase the biomass and lipid content of microalgae. Park et al. [65] compared the economic viability of the phytohormones on biomass production with normal synthetic medium and acetate. The cost of biomass production with plant hormones was 0.014 US$/g which was cheaper and more economical than the production of biomass without plant hormones and with acetate, which were 0.024 US$/g and 0.017 US$/g, respectively. In another study, IAA and DAH plant hormones induced the biomass production considerably which cost about 0.39 US$/g and 0.30 US$/g, respectively, which is more economical than using the normal media without plant hormone (0.78 US$/g). In the present study, plant hormones were shown to increase biomass and lipid content of *Chlorella* sp. with more advantage on economical aspect.

## Conclusion

In the present study, the different plant hormones were used to enhance the growth of *Chlorella* sp. and its lipid content enriched in EPA and DHA. Four different plant hormones affected *Chlorella* sp. differently with respect to the biomass and the lipid content. The *Chlorella* sp. treated with IAA at 1 mg/L showed an increase in biomass, lipid content and EPA and DHA production. The addition of plant hormones improved ROS content and the cell responded against ROS by increasing antioxidant enzymes activities. The ROS-induced oxidative stress improved biomass and lipid content as well as upregulating the fatty acid biosynthesis genes. Treatment with IAA specifically upregulated *fad* which is responsible for the increased omega-3 fatty acids. EPA and DHA esters are acceptable form of drug for the severe hypertriglyceridemia. Among the four plant hormones tested, ABA-treated *Chlorella* sp. can be utilized for biodiesel applications and IAA-treated *Chlorella* sp. had the highest contents of EPA and DHA which are important for several medical applications. The isolated *Chlorella* sp. responded differently with different plant hormones and efficiently increased the biomass and lipid content upon treatment with low concentration of plant hormone.

## Methods

### Microorganism and culture conditions

In this study, *Chlorella* sp. isolated from the stone quarry pond water was used; its obtained accession number was KP972095 from GenBank [66]. The culture was maintained and grown in BG11 medium with 100 rpm shaking at 27 ± 1 °C under continuous illumination of 50 μmol photons/m^2^/s. The purity of the culture was monitored routinely by microscopic observation.

### Hormone treatment

The hormone and concentration range were chosen based on the preliminary investigations and previously reported studies [10, 67]. All the hormones were obtained from Sigma Aldrich (USA). The hormone treatment experiments were conducted in BG11 medium supplemented with different hormones independently. The hormones used were zeatin, IAA, GBA and ABA, with the concentration range of 0.5–2 mg/l, except for zeatin where the concentration range was 0.05–0.2 mg/l. The initial cell density was set as OD_730_ ∼ 0.05. The cells were grown for 12 days and used for further investigations.

### Biomass and lipid determination

The biomass content analysis was done by dry cell weight (DCW) determination method [69]. The grown cell was collected by centrifugation at 2790*g* for 10 min and the cell pellet was lyophilized using freeze dryer for the DCW analysis. The lipid extraction was done by solvent extraction method as described by Sivaramakrishnan and Incharoensakdi [68].

### Growth rate measurement

The growth rate of microalgae was determined as described by Kong et al. [69]. The growth curve was calculated using Eq. 1:

1 $$\mu_{{max} } = \, \ln \, \left( {x/x_{0} } \right)/t_{2} - t_{1} .$$

µ is the growth rate, whereas x is DCW at the end of exponential phase at time t_2_ and x_0_ is DCW at the initial exponential phase at time t_1_.

The doubling time of algal biomass was determined by using the equation as described by Mulumba and Farag [70] using Eq. 2:

2 $$t_{d} = \ln \left( 2 \right)/\mu .$$

### H_2_O_2_, malondialdehyde and antioxidant enzyme determination

The H_2_O_2_ content of algal cells were determined by treating the cells with 1 M potassium iodide and the absorbance was read spectrophotometrically at 390 nm. The values were determined from the standard using fresh H_2_O_2_ solutions [71]. The MDA content was determined by mixing 1 ml of 0.5% thiobarbituric acid in 20% trichloroacetic acid and 0.5 ml supernatant with vortex mixing. The mixture was kept 15 min in boiling water bath and the cooled mixture was centrifuged at 2790 g for 10 min. The absorbance was read at 450, 532 and 600 nm and the values were used in the following equation to determine the MDA content [72]:

3 $${\text{MDA }}\left( {\upmu{\text{mol}}/{\text{g fresh weight}}} \right) \, = \, \left[ {6.45 \, * \, \left( {{\text{OD}}_{532} - {\text{ OD}}_{600} } \right)} \right] - \left[ {0.56{\text{ OD}}_{450} } \right]/{\text{fresh weight }}\left( {\text{g}} \right).$$

The SOD and CAT activities of hormone-treated lysed cell suspension were determined by WST-1 reagent (Sigma Aldrich, USA) protocol and CAT assay kit (Sigma Aldrich, USA) protocol, respectively.

### Fatty acid analysis

The determination of lipid composition analysis was done according to Sivaramakrishnan and Incharoensakdi [73].

### Statistical analysis

The values obtained from the mean of triplicate experiments and the standard deviation values are shown as error bars (mean ± SD, *n* = 3). The GraphPad software was used for *t* test comparisons to analyze the statistical significance (*p *< 0.05).

## Supplementary information

**Additional file 1: Table S.1.** The fuel properties of ABA treated *Chlorella* sp. **Table S.2.** Primers used. **Fig. S.1.** The *Chlorella* sp. observed under a light microscope at 40X magnification. **Fig. S.2.a.b.c.** Response of Chlorophyll a, carotenoids and oxygen evolution rate after plant hormone treatment. **Fig. S.3.** FTIR spectra of control and plant hormone (optimized only) treated *Chlorella* sp. **Fig. S.4.** The genes involved in the omega-3 fatty acid synthesis pathway.

## Data Availability

The data of this study are included in this article and its additional file.

## Abbreviations

ABA: abscisic acid
*acp*: acyl-acyl carier protein
accD: acyl CoA carboxylase D
CAT: catalase
DAH: diethyl amino ethyl hexanoate
DCW: dry cell weight
DHA: docosahexaenoic acid
EPA: eicosapentaenoic acid
*fata*: acyl carrier protein thioesterase
GBA: gibberellic acid
IAA: indole acetic acid
PEPC: phosphoenol pyruvate carboxylase
PUFA: polyunsaturated fatty acids
*mctk*: malonyl CoA:ACP transacylase
MDA: malondialdehyde
*fad*: omega-3 fatty acid desaturase
ROS: reactive oxygen species
SOD: superoxide dismutase
