# Supplementary material for: Plant hormone induced enrichment of Chlorella sp. omega-3 fatty acids
Source: Biotechnol Biofuels. 2020 Jan 17;13:7. doi: 10.1186/s13068-019-1647-9 (PMC6966795; doi:10.1186/s13068-019-1647-9)
Supplement: Supplementary file 1 — Additional file 1: Table S.1. The fuel properties of ABA treated Chlorella sp. Table S.2. Primers used. Fig. S.1. The Chlorella sp. observed under a light microscope at 40X magnification. Fig. S.2.a.b.c. Response of Chlorophyll a, carotenoids and oxygen evolution rate after plant hormone treatment. Fig. S.3. FTIR spectra of control and plant hormone (optimized only) treated Chlorella sp. Fig. S.4. The genes involved in the omega-3 fatty acid synthesis pathway. [file 13068_2019_1647_MOESM1_ESM.docx]

**Additional file for**

**Plant hormone induced enrichment of *Chlorella* sp. omega fatty acids for ethyl ester production**

**Ramachandran Sivaramakrishnan, Aran Incharoensakdi***

**Laboratory of Cyanobacterial Biotechnology**

**Department of Biochemistry**

**Faculty of Science**

**Chulalongkorn University**

**Bangkok 10330, Thailand**

***Corresponding Author**

**E-mail:** [**aran.i@chula.ac.th**](mailto:aran.i@chula.ac.th)

**Phone No: +66 2 218 5422**

**Fax No: +66 2 218 5418.**

**S.1 *Chlorella sp.* used in this study.**

The *Chlorella* sp. was observed under a light microscope at 40X magnification


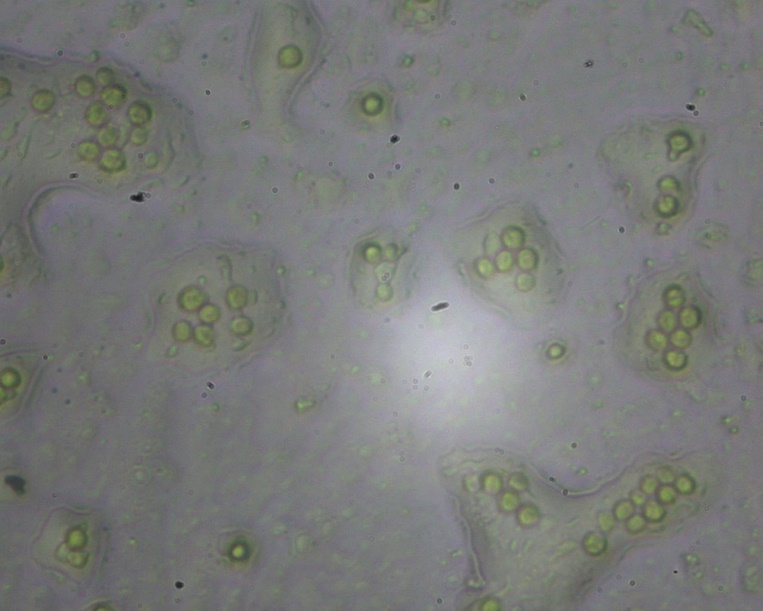


**Fig. S.1**

**S.2 GC analysis**

The transesterification product was analyzed using Agilent gas chromatograph (GC) (Santa Clara, California, USA) equipped with the carbowax column (Santa Clara, California, USA) and flame ionization detector (FID). The carrier gas used was N_2_ with a flow rate of 1 ml/min. H_2_ and O_2_ were used for the ignition purpose. The initial temperature of column was 150 °C and increased at a rate of 10 °C/min up to 240°C and maintained the same temperature for 10 min. The temperature of injector and detector were set as 250 °C. The 10 μl of sample was mixed with an internal standard of 230 μl (methyl heptadecanoate) and ester content was determined according to the EN14103 (European Normalization) method (Sivaramakrishnan and Incharoensakdi, 2016.

The methyl ester yield was calculated using the formula,

$$methyl ester yield \left( \% \right)=\frac{Weight of oil methyl ester \left( g \right)}{Weight of lipid \left( g \right)}X100$$

**S.3** **Response of Chlorophyll a, carotenoids and oxygen evolution rate after plant hormone treatment**

The levels of Chl a content were shown in Fig.S.2.a The Chl a level was elevated at 0.1 mg/L for zeatin and 1 mg/L for other hormones. Increasing hormone concentration than the optimal level decreases Chl a content in all cases. Plant hormones like auxins improves the plant growth and which enhances the chlorophyll content by stimulating the photosynthesis and chlorophyll activates the cellular redox systems [1]. Auxins improves both Chlorophyll a and b content and carotenoid content of *C.pyrenoidosa* [2]. Improving chlorophyll content increasing the light capturing capacity which improves the various biomolecules content in the cells, especially lipids [3].

As like chlorophyll, carotenoids content also was improved and the results were shown in Fig.S.2.b. At optimized growth condition for all the hormone treated cell showed higher carotenoid content. Carotenoid content of all the hormone treated cells showed increase which is higher than the control. Plant hormone induced carotenoid accumulation in C.pyrenoidosa ZF strains [4]. The plant hormone addition increased the carotenoid content significantly after 12 days of cultivation. Carotenoids plays an important role in accessory light-harvesting pigments, carotenogenesis was induced by the plant hormonones [5].

Plant horomones induces the oxygen evolution rate in all optimized growth conditions (Fig.S.2.c). The light harvesting complex in the chlorophylls (photosystem) captures the light energy and it is move for photochemistry and thus oxygen evolution occurs [6]. In this study, chlorophyll content was high as compared to the control. However, the oxygen evolution rate is not increased in all cases particularly with Gibberellic acid treatment. IAA and ABA showed highest oxygen evolution rate at optimized growth conditions. Zeatin responds moderately as compared to the control. In the present study, IAA and ABA regulates the photosynthesis well than the other two hormones.


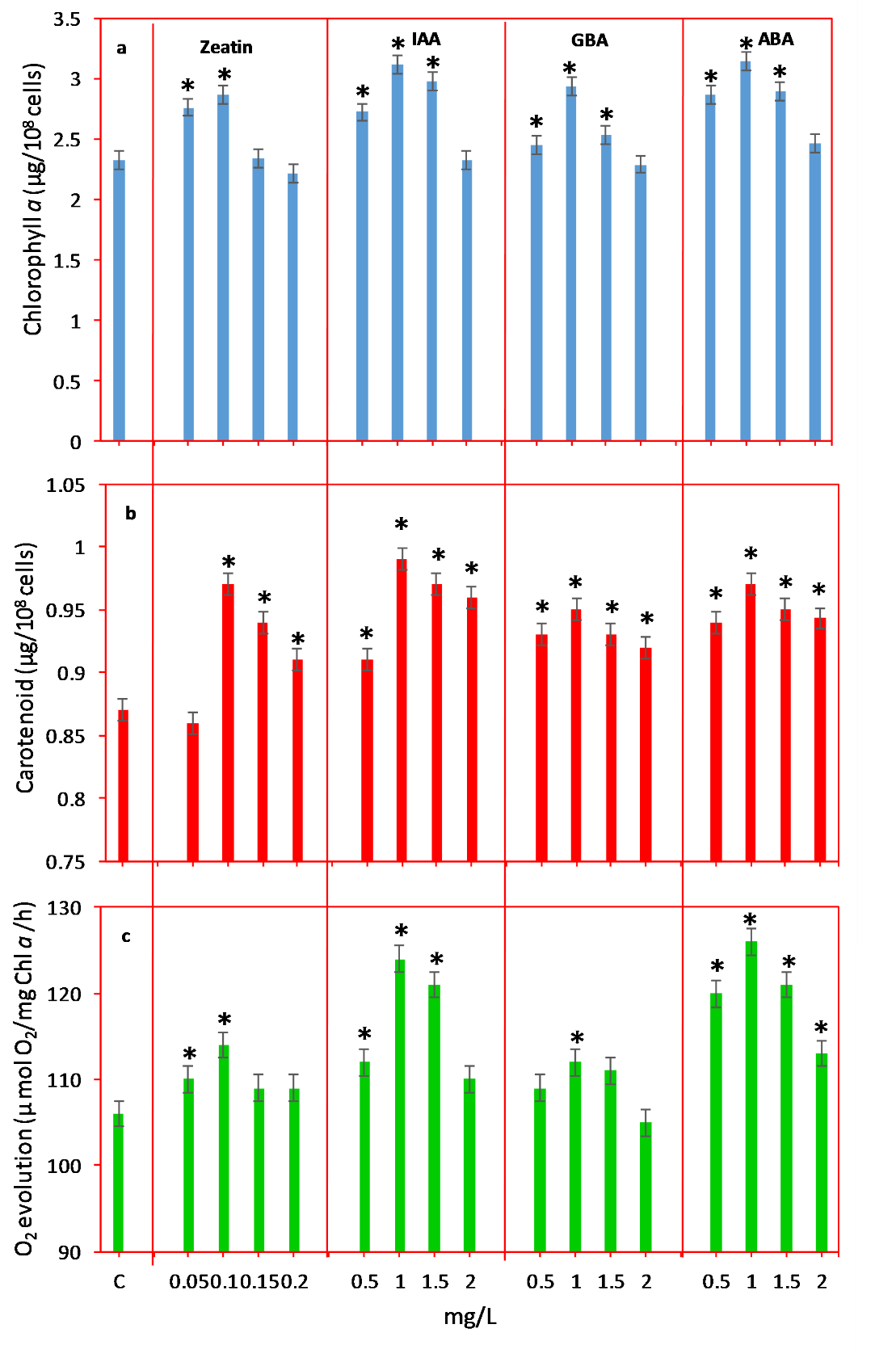


**Fig. S.2.a.b.c**

**S.4 FTIR spectra of control and plant hormone (optimized only) treated Chlorella sp.**

**Method**

For FTIR spectrum analysis Perkin-Elmer infrared spectrophotometer was used. The samples (dry biomass) was pelleted using KBr (potassium bromide) with the 10-13 mm diameter and 1mm thickness. Spectra were recorded in transmission mode with the spectral region of 4000 – 500 Cm^-1^ [7].

**Results**

The FTIR spectrum of treated and untreated Chlorella sp. were shown in Fig. The signals between 3025-2954 cm^-1^ and 1746-1654 cm^-1^ were belong to the lipid functional groups [7]. In the present study the stretches at 2960 and 1696 cm^-1^ clearly showing the variations upon hormone addition. The stretches were at this point are clearly visible with IAA and ABA. The signal at 1696 cm^-1^ belong to ester group (C=O). The other signal 1460 cm^-1^ belong to CH_2_ bending and stretches between 1070-1250 cm^-1^ corresponds to C-O-C stretching in ester groups and all these signals are the principle indication of the lipid groups [8]. The stretches between 3700- 3000 and 1150-1030 cm^-1^ are indicating the carbohydrate groups. The signals between 1655 and 1548 cm^-1^ are belongs to protein content. In the present the signal at 1605 showed variations upon after the plant hormone treatment which indicates the changes in proteins. Carotenoid is also a protein the changes in the carotenoid is notable after the plant hormone treatment.


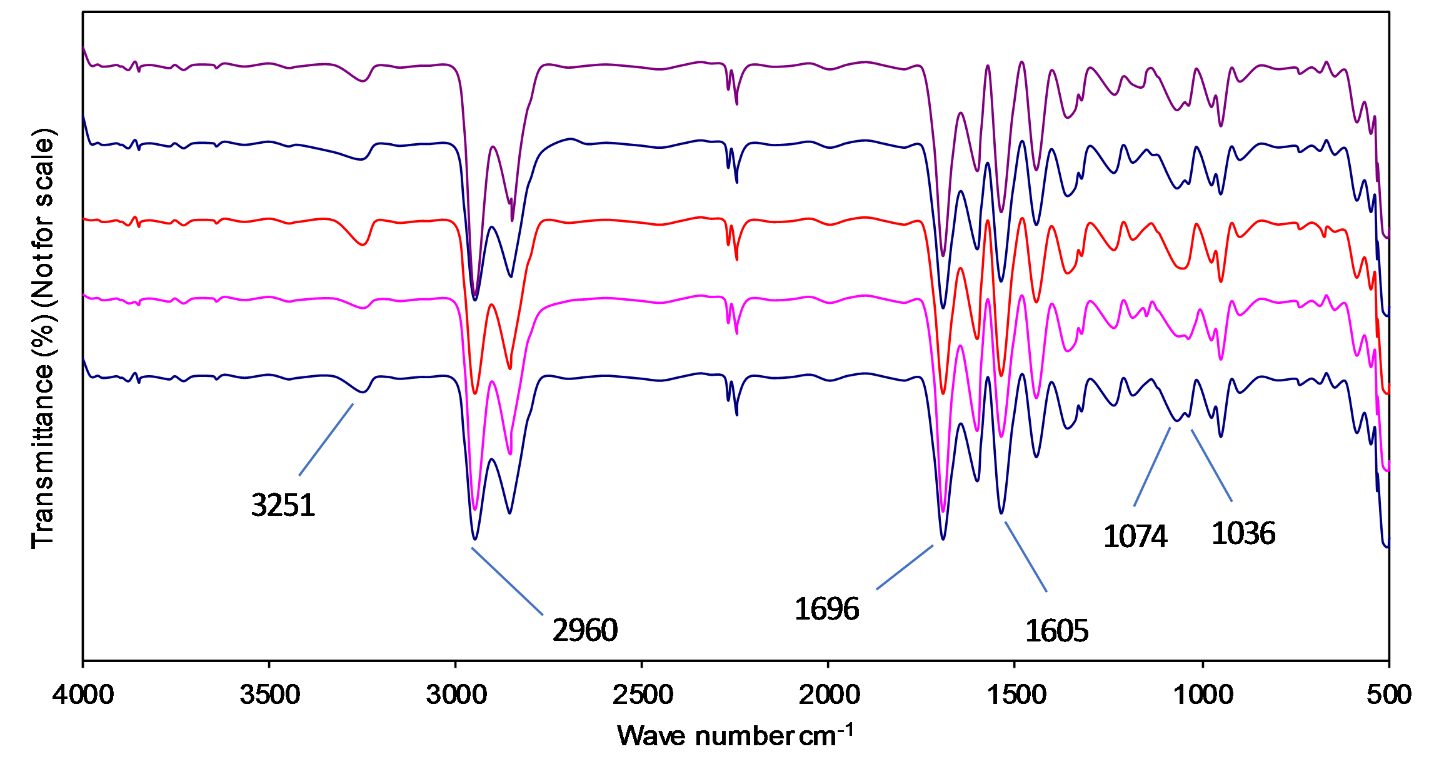


**Fig. S.3**

**S. 5 The fuel properties of ABA treated cells**

The fuel properties were analyzed as mentioned in our previous study [9]. The fuel properties were determined from fatty acids obtained after ABA treatment and shown in Table S.1. The values were compared with the standards (American society for testing and materials - ASTM D6751). The cetane number is the lead quality of biodiesel and it is ignition delay time which is directly involved in the combustion mechanism [10]. The ABA treated *Chlorella* sp. showed the cetane value greater than 47 (>47) and comply with the standards. The smoke emitted from the engine will be high if the cetane value is high. Iodine value (IV) is another prime quality of biodiesel and it is an amount of total unsaturation and it signifies the oxidative stability of biodiesel [11]. The IV value should be lower than 120, all the ABA treated *Chlorella* sp. showed acceptable values as standards. Degree of unsaturation (DU) depicts the long-term storage stability and it is calculated from mono and poly unsaturated fatty acids content [10]. The DU values obtained for ABA treated *Chlorella* sp. were close to those reported by Wu and Miao [10].

**Table S.1**

The fuel properties of ABA treated *Chlorella* sp.

|  |  |  | ABA concentration mg/L | | | |
| --- | --- | --- | --- | --- | --- | --- |
| Properties | EN | ASTM | 0.5 | 1 | 1.5 | 2.0 |
| SV | NA | NA | 208.72 | 208.31 | 208.22 | 208.26 |
| IV | 120 | NA | 110.8 | 106.45 | 114.38 | 116.98 |
| CN | >51 | >47 | 47.52 | 48.55 | 47.08 | 47.01 |
| DU | NA | NA | 103.31 | 101.6 | 106.96 | 108.9 |

Units for properties: SV (mg KOH g−1); IV (g I2 100 g−1); CN (> 47); DU (wt %).

EN – European standards, ASTM – American society for testing and materials;

NA – not available.

**Transcriptional expression level of genes involved in the fatty acid biosynthesis**

The control and the optimum conditions of plant hormone treated samples were taken for the RT-PCR analysis. The microalgal total RNA was extracted and cDNA was synthesized as mentioned in the Sivaramakrishnan and Incharoensakdi, 2018. The gene sequences were retrieved from the NCBI database for HM560036, HM56034, HM560037, HM56034, HM56035 for acyl-acyl carrier protein (*acp*), malonyl-CoA:ACP transacylase (*mctk*), acyl carrier protein thioesterase (*fata*) and omega-3 fatty acid desaturase (*fad*) respectively and scheme was shown in Fig.S.4 (modified from Lei et al. [12]) and the primers were designed according to the Lei et al. [12] (Table S.2) 16s rRNA gene was used as a control and the PCR conditions are: initial denaturation 94 °C for 4 min followed by 27 cycles of denaturation at 94 °C for 30 s, annealing at 55 °C for 1 min and final extension at 72 °C for 3 min. A 1% agarose gel electrophoresis was used to analyze the PCR products. Gel analyzer 2010a software was used in the analysis of band intensities of cDNA of different genes 16s rRNA to determine the gene expression levels. The acetyl-CoA carboxylase activity was determined according to the method described in Sivaramakrishnan and Incharoensakdi [13]


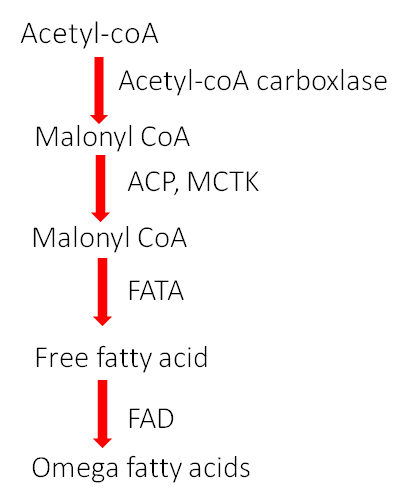


**Fig. S.5**

**Table S.3**

| Genes used | Primers | Product length |
| --- | --- | --- |
| ACP | F- CAGCTCGGCACTGACCTTG | 120 |
|  | R- CAAGGGTCAGCTCGAACTTCTC |  |
| MCTK | F- GGTGAGGACAAGGCGGTG | 120 |
|  | R- TCATCCTGGCCTTGAAGCTC |  |
| FATA | F- AGACTCGTTCAGCGAGGAGC | 120 |
|  | R- CATGCCCACAGCATGGTTC |  |
| FAD | GTAGGTCACCACGTCCAGCC | 120 |
|  | CTTGATAGGCATGCTGGGTGT |  |

**References**

[1] Du H, Ahmed F, Lin B, Li Z, Huang Y, Sun G, Ding H, Wang C, Meng C, Gao Z. The effects of plant growth Regulators on cell growth, protein, carotenoid, PUFAs and lipid production of *Chlorella pyrenoidosa* ZF Strain. Energies. 2017;10:1696, 1-23.

[2] Kobayashi M, Todoroki Y, Hirai N, Kurimura Y, Ohigashi H, Tsuji Y. Biological activities of abscisic acid analogs in the morphological change of the green alga *Haematococcus pluvialis*. J Ferment Bioeng. 1998;85:529–531.

[3] Chokshi K, Pancha I, Ghosh A, Mishra S. Salinity induced oxidative stress alters the physiological responses and improves the biofuel potential of green microalgae *Acutodesmus dimorphus*. Bioresour Technol. 2017;244:1376–1383.

[4] Piotrowska-Niczyporuk A, Bajguz A. The effect of natural and synthetic auxins on the growth, metabolite content and antioxidant response of green alga Chlorella vulgaris (Trebouxiophyceae). Plant Growth Regul 2014:73:57–66.

[5] Czerpak R, Bajguz A, Białecka B, Wierzchołowska LE, Wolanska MM. Effect of auxin precursors and chemical analogues on the growth and chemical composition in *Chlorella pyrenoidosa* Chick Acta Soc Bot Pol. 1994;63:279–286.

[6] Oey M, Ross IL, Stephens E, Steinbeck J, Wolf J, Radzun KA, et al. RNAi knock-down of LHCBM1, 2 and 3 increases photosynthetic H2 production efficiency of the green alga *Chlamydomonas reinhardtii*. PLoS ONE. 2013;e61375, 1-12

[7] Yu C, Irudayaraj J. Spectroscopic characterization of microorganisms by Fourier transform infrared microspectroscopy. Biopolymers. 2005;77:368-377.

[8] Forfang K, Zimmermann B, Kosa G, Kohler A, Shapaval V. FTIR spectroscopy for evaluation and monitoring of lipid extraction efficiency for oleaginous Fungi. PLoS ONE. 2017;0170611:1-17

[9] Sivaramakrishnan R, Incharoensakdi A. Enhancement of total lipid yield by nitrogen, carbon, and iron supplementation in isolated microalgae. J Phycol. 2017;53:855–68.

[10] Wu H, Miao X. Biodiesel quality and biochemical changes of microalgae *Chlorella pyrenoidosa* and *Scenedesmus obliquus* in response to nitrate levels. Bioresour Technol. 2014; 174:421–7.

[11] Knothe G. Improving Biodiesel Fuel properties by modifying fatty Esters composition. Energy Environ Sci. 2009;2:759–66.

[12] Lei AP, Chen H, Shen GM, Hu ZL, Chen L, Wang JX. Expression of fatty acid synthesis genes and fatty acid accumulation in *Haematococcus pluvialis* under different stressors. Biotechnol Biofuels 2012;5:18–28.

[13] Sivaramakrishnan R, Incharoensakdi A. Enhancement of lipid production in *Synechocystis* sp. PCC 6803 overexpressing glycerol kinase under oxidative stress with glycerol supplementation. Bioresour Technol. 2018;267:532–540.
